# Production of Protease Inhibitor With *Penicillium* sp. — Optimization of the Medium for Growth in Pellet Form and Cytotoxicity Testing

**DOI:** 10.1002/elsc.70012

**Published:** 2025-03-17

**Authors:** Winda Soerjawinata, Shila Prajapati, Isabelle Barth, Xiaohua Lu, Roland Ulber, Thomas Efferth, Percy Kampeis

**Affiliations:** ^1^ Environmental Campus Birkenfeld Institute for Biotechnical Process Design Trier University of Applied Sciences Birkenfeld Germany; ^2^ Institute of Pharmaceutical and Biomedical Sciences Johannes Gutenberg University Mainz Mainz Germany; ^3^ Institute of Bioprocess Engineering University of Kaiserslautern‐Landau (RPTU) Kaiserslautern Germany

**Keywords:** cytotoxicity testing, fungal cultivation, fungal growth, pellet morphology, protease inhibitor

## Abstract

*Penicillium* sp. (IBWF 040‐09) produces a protease inhibitor that can potentially be used against the main protease of human African trypanosomiasis. Since the target substance is formed intracellularly (under nutrient limitation), the fungal pellet is preferred compared to the free mycelia in bioreactor cultivation. The optimization of the production of protease inhibitor became the main focus of this study. The effects of the concentrations of spores, calcium chloride, and Pluronic F68 were investigated with regard to fungal growth, pellet morphology, and the production of protease inhibitor. The combination of adjusting the spore concentration and adding Pluronic F68 and calcium chloride increased the probability of achieving the desired morphology. This ensured better reproducibility of the production of the target substance by *Penicillium* sp. (IBWF 040‐09) with the bioreactor system used. In addition, the protease inhibitor was tested in a resazurin assay and showed no noticeable cytotoxic effects on peripheral blood mononuclear cells isolated from whole blood cells.

**Abbreviations**
ACN: acetonitrile
BDM: bio dry mass
HAT: human African trypanosomiasis
PBMCs: peripheral blood mononuclear cells
YMG: yeast extract, malt extract, glucose

## 1 Introduction

The macromorphology of filamentous microorganisms is of great interest, since it strongly influences the productivity of target substances [1, 2, 3, 4]. Adjustments of cultivation parameters are required for different morphologies [5, 6, 7, 8, 9]. Cultivations have been optimized to obtain desired forms (either dispersed or clumped/aggregated) in order to improve the space‐time yield of the target substance. In general, spores are categorized into two types: non‐coagulative and coagulative. Non‐coagulative spores form a single pellet from a single spore, whereas coagulative spores form one pellet from multiple spores. However, the basis of this classification cannot be attributed to a single factor, as it varies even at the strain level [10]. For example, the spores of *Penicillium chrysogenum* were classified by Nielsen et al. [11] as non‐coagulative. In their study, the spores of *P. chrysogenum* germinated and formed clumps. These clumps developed into denser pellets once a certain hyphal length was achieved. In contrast, Veiter et al. [12] categorized *P. chrysogenum* into a different type: the hyphal element agglomerating type. Depending on the cultivation conditions, *P. chrysogenum* exhibited characteristics of both coagulative and non‐coagulative types. Another spore‐forming fungus, *Aspergillus niger*, is an example of a coagulative type. Grimm et al. [13] had elucidated the mechanism of pellet formation of *A. niger* in detail. In their study, the inoculated spores went through two stages of the aggregation process. The first one occurred quickly after the inoculation and was barely influenced by hydrodynamic forces [14]. During the second aggregation, germination also occurred and thus, the aggregation took place not only between spores but also between hyphae and between hyphae and spores. Various environmental factors have an impact on the aggregation processes, such as physical factors (e.g., the addition of multivalent cations and surface‐active agents) and biological factors (e.g., the concentration of spores in the inoculum) [15]. The rate of the second aggregation process rises with increasing spore concentration. Furthermore, the spore concentration also influences the pellet amount as well as the pellet size. A low spore concentration tends to result in a low number of pellets. In this case, however, the diameters of the pellets are larger than those of the pellets from cultivations with higher spore concentrations [13].

**Summary**

- This study offers the possibility of increasing the reproducibility of the production of target substances from shear‐sensitive filamentous fungi, particularly if the pellet form is desired for the formation of intracellular substances.
- For that purpose, the hydrodynamic shear stress in the bioreactor cultivation can be reduced by adding Pluronic F68.
- In addition, pellet formation during the lag phase can be supported by calcium chloride.
- The combination of both substances leads to a well‐preserved pellet shape until the end of the cultivation and, above all, to a higher probability of the production of an intracellular substance such as protease inhibitor from *Penicillium* sp. (IBWF 040‐09).

As bivalent cations, calcium ions induce cell aggregation by overcoming electrostatic repulsion, especially at pH values above 5.5, where most fungal cells are negatively charged [16]. Pera and Callieri [17] showed an increase in citric acid production by *A. niger* in the presence of Ca^2+^, which also led to more branched hyphae and bulky cells. Several surfactants have been investigated in the cultivation of filamentous microorganism. Vecht‐Lifshitz et al. [18] reported the influence of Triton X‐100, Pluronic F68, as well as Brij 58 on the pellet formation of filamentous *Streptomyces tendae* by inducing the interaction between the hyphae. Pluronic F68 is particularly known as non‐ionic surfactant that is often used in the cultivation of shear‐sensitive mammalian cells. Pluronic F68 provides cellular protection from hydrodynamic stress by reducing the bubble velocity, stabilizing the bubbles, and decreasing interactions between cells and bubbles [19, 20]. In addition, Pluronic F68, at optimum concentrations, even optimizes oxygen mass transfer [20].

Since the tendency of pellet formation is different for each type of filamentous microorganism, the effects of spore concentration and the addition of calcium chloride (CaCl_2_) and Pluronic F68 on the growth of *Penicillium* sp. (IBWF 040‐09) were investigated in this study. Particular attention was paid to the extent of formation of a protease‐inhibiting secondary metabolite produced intracellularly by *Penicillium* sp. (IBWF 040‐09). This substance could be a potential drug against human African trypanosomiasis (HAT). However, this may be only possible if the substance is not toxic to human cells. Accordingly, cytotoxicity tests of this substance against human blood cells were carried out in parallel with the optimization of the cultivation conditions.

## 2 Materials and Methods

### 2.1 Strain and Inoculum Preparation

*Penicillium* sp. (IBWF 040‐09) was maintained as described in the study by Soerjawinata et al. [21]. The preparation of the spore solutions for inoculation is also described there. The spore concentration for bioreactor cultivation was varied at 4.41 · 10^3^ Sp/L, 4.41 · 10^5^ Sp/L, and 4.41 · 10^7^ Sp/L. The desired spore concentration in the bioreactor was achieved by adjusting the number of spores in the inoculum accordingly. The volume of the inoculum solution was 1–5 mL and can, therefore, be neglected in relation to the 2 L filling volume of the bioreactor.

### 2.2 Cultivation Conditions

All bioreactor cultivations presented were performed in a glass bioreactor UniVessel Glass DW 2 L from Sartorius Stedim Biotech GmbH (Göttingen, Germany). The equipment and geometry of the bioreactor are described in [21] and [22]. YMG medium with the composition described in [21] was used for the cultivations, with additional 25 µL/L polypropylene glycol as an antifoam agent. The concentrations of CaCl_2_ (0 g/L, 0.5 g/L, 1.0 g/L, and 2.0 g/L) from Carl Roth GmbH & Co. KG (Karlsruhe, Germany) and Pluronic F68 (0 % (w/v) and 0.2 % (w/v)), also known as Kolliphor P 188, from Merck KGaA (Darmstadt, Germany) were chosen to investigate the bioreactor cultivation. The addition of these substances was carried out through sterile filters after autoclave sterilization of the bioreactor filled with YMG medium. The pH value of the medium was adjusted to 5.5 after all substances were added using sterile‐filtered 1 M NaOH. The bioreactor cultivations were carried out at 22°C with a constant stirring speed of 350 rpm (refers to a tip speed of 1.28 m/s). The aeration with atmospheric air was initially started with a constant flow of 1 L/min (corresponds to 0.5 vvm). The dissolved oxygen (DO) controller was started as soon as DO saturation reached a value below 30 %. The volume flow of the atmospheric air was then increased up to 2.25 L/min. If necessary, up to 0.25 L/min of pure oxygen was added to keep the DO value at 30 %. The cultivations were ended if the aeration rate decreased and went back to 1 L/min. During the cultivations, samples were taken regularly for further analysis of bio dry mass (BDM), substrate concentration (glucose and maltose), pellet macro‐ and micro‐morphology analysis, as well as production of protease inhibitor.

### 2.3 Determination of Biomass Concentration and Growth Rate

Biomass concentration was determined by two methods, online and offline. Online monitoring of biomass concentration was carried out using a non‐invasive sensor CGQ BioR from Scientific Bioprocessing (Baesweiler, Germany) that was attached to the reactor wall. DOTS Software from Scientific Bioprocessing was used to collect the data recorded by the sensor. This sensor uses the backscatter light method to measure the cell density. The proportionality between sensor signal and biomass concentration was previously explained in [22]. On the other hand, the gravimetric method was also performed to determine the biomass concentration offline. For this purpose, the samples were filtered using pre‐dried and pre‐weighted 1.5 µm glass microfiber filter 696 with a diameter of 47 mm from VWR International GmbH (Darmstadt, Germany). The filter cakes were put in an oven UT12P from Thermo Electron LED GmbH (Langensbold, Germany) at 80°C for 48 h. Based on the biomass concentration determined, the average growth rates (*μ*, expressed in h^−1^) were calculated according to [21].

### 2.4 Extraction and Chromatographic Separation of Protease Inhibitor

After cultivation, the fungal biomass was harvested, homogenized, and extracted. The protease‐inhibiting substance was separated using a chromatography system equipped with a fraction collector for 96‐well plates. Extraction and chromatographic separation were performed as explained in [21], with the modification of UV detection at a wavelength of 210 nm. After the chromatographic separation, the protease‐inhibiting substance was located in well B6 of the 96‐well plates. In addition, a larger scale chromatographic separation was also carried out in this study using the 1290 Infinity II Preparative LC system from Agilent Technologies Inc. (Santa Clara, CA, USA) with the column InfinityLab Pursuit XRs C18 preparative high‐performance liquid chromatography (HPLC) column (30 × 250 mm, 5 µm) also from Agilent Technologies Inc. The mobile phases used were acetonitrile (ACN) with 0.1 % formic acid and water with 0.1 % formic acid, with a flow rate of 45 mL/min. Gradient elution was carried out according to the following procedure: 0–14.99 min isocratic water–ACN with 1 % ACN, 15–24.99 min isocratic water–ACN with 6 % ACN, and 25–30 min isocratic 100 % ACN. The column was tempered to 40°C. The UV detection was carried out at a wavelength of 210 nm. The injection volume was 2.5 mL, containing 100 mg extract. Using this chromatography method, the target substance was located in the fraction, which was collected between 18.0 min and 20.8 min. The HPLC fractions were dried at room temperature to be used for bioassay (see Section 2.5) and toxicity assay (see Section 2.6).

### 2.5 Protease Inhibition Assay

The protease rhodesain used for the inhibition assay in this study was expressed and purified by the working group of Prof. Dr. Tanja Schirmeister as published in [23]. The measurement of the increase in fluorescence signal upon cleavage of the fluorogenic substrate N‐Cbz‐Phe‐Arg‐7‐amido‐4‐methylcoumarin hydrochloride (N‐Cbz‐Phe‐Arg‐AMC·HCl) from Bachem Holding AG (Bubendorf, Switzerland) by rhodesain was carried out using a microplate reader Fluoroskan Ascent (λ_ex_ = 365 nm, λ_em_ = 460 nm) from Thermo Scientific Inc. (Dreieich, Germany). Assays were carried out in black flat‐bottom 96‐well microtiter plates Rotilabo from Carl Roth GmbH & Co. KG. The detailed implementation of the assay is described in [21]. The protease‐inhibiting activities of B6 fractions from analytical HPLC and the corresponding fractions from preparative HPLC (see Section 2.4) were measured in two independent measurements.

### 2.6 Cytotoxicity Assay

The cytotoxicity assay was performed by determining the cell viability after treating healthy cells with the fraction from preparative HPLC, which contained the protease inhibitor (see Section 2.4). Peripheral blood mononuclear cells (PBMCs) were isolated from whole blood from healthy donors using Histopaque‐1077 from Sigma‐Aldrich Co. LLC (St. Louis, MO, USA), according to the manufacturer's instructions. Isolated PBMCs were resuspended in Panserin 413 medium from PAN‐Biotech (Aidenbach, Germany), supplemented with 2 % phytohemagglutinin M from Life Technologies (Darmstadt, Germany) and then incubated in a humidified atmosphere of 5 % CO_2_ at 37°C. PBMCs, at a concentration of 10^4^ cells, were treated with the above‐mentioned HPLC fraction dissolved in DMSO from VWR International GmbH. A serial dilution of the cell suspension was prepared: 10 mg/mL, 3 mg/mL, 0.9 mg/mL, 0.27 mg/mL, 0.081 mg/mL, 0.024 mg/mL, 0.007 mg/mL, and 0.002 mg/mL. A mixture of ACN and water with the ratio of 1:1 was used as negative control. The pre‐treated cells were incubated for 72 h at 37°C. Afterward, resazurin assay was performed by adding 20 µL of 0.01 % (w/v) resazurin from Sigma‐Aldrich Co. LLC into the cell suspension, followed by an incubation for 4 h at 37°C. The fluorescence intensity was measured using the Infinite M200 Pro plate reader (λ_ex_ = 544 nm, λ_em_ = 590 nm) from Tecan Trading AG (Männedorf, Switzerland).

### 2.7 Preparation of Thin‐Cut Fungal Pellets

The pellets from the bioreactor cultivations were transferred to embedding cassettes and washed twice using 0.01 M phosphate‐buffered saline (PBS; pH = 7.4). The pellets were first incubated for 1 h in PBS containing 20 % (v/v) lactophenol blue from Sigma‐Aldrich Co. LLC and then rinsed twice using PBS. This was followed by a second incubation for 3 h in 4 % (v/v) glutaraldehyde from Merck KGaA and two further rinses with PBS. Subsequently, a dehydration process was carried out by several incubations in isopropanol–water mixtures with increasing isopropanol concentrations (25 %, 50 %, 70 %, 80 %, 95 %, and 100 %). The used isopropanol from VWR International GmbH had a purity of 99.7 %. The incubation time in 25–95 % isopropanol was 10 min in each case. The final dehydration in 100 % isopropanol was carried out twice consecutively: first for 20 min and then for 25 min. The pellets were then put into a mixture of isopropanol and ROTI Histol (1:1) from Carl Roth GmbH & Co. KG for 10 min. The final clearing process was done in 100 % ROTI Histol for another 10 min. All steps of the previously described incubation process were carried out at room temperature, with 200 rpm shaking speed. The pellets were then incubated in melted paraffin ROTI Plast from Carl Roth GmbH & Co. KG for 40 min before the paraffin block was solidified on a cooling plate MPS C from SLEE medical GmbH (Nieder‐Olm, Germany). The embedded pellets were cut in 20 µm slices using a microtome CUT 6062 from SLEE medical GmbH. These slices were then deparaffinized for microscopic examination. Deparaffinization was started by melting the paraffin at 65°C for 1 h. A rehydration process was carried out by several incubations in isopropanol–water mixtures with decreasing isopropanol concentrations (100 %, 90 %, 80 %, and 70 %) for 15 min each. Final rehydration was done in distilled water for 20 min. Afterward, the slices were colored using lactophenol blue for 15 min. Then, the colored slices were covered using water‐free covering agent Neo‐Mount from Merck KGaA. For this purpose, the samples had to be dehydrated again with isopropanol–water mixtures with increasing isopropanol concentrations (70 %, 80 %, 90 %, and 100 %) for 2 s each.

### 2.8 Morphology Analysis of Fungal Pellets

Thin‐sliced pellets (according to Section 2.7) were observed using an Olympus IX 71 inverted microscope (Evident Europe GmbH, Germany), which was equipped with a PlanC N 10X objective lens and an Olympus XC10 camera (Evident Europe GmbH). Olympus cellSens Standard 4.1 software (Evident Europe GmbH) was used to acquire images of the thin‐cuts. This software was also used to measure the outer diameters of the thin‐cuts, regardless of whether the thin‐cut of the pellet was completely filled with biomass. The circularities of the thin‐sliced pellets were further analyzed using the open‐source software FIJI [24]. With this software, the images were converted into 8‐bit format before thresholds were applied. A “Mean” threshold (160–225) with automatic adjustment was used for all images analyzed in this study. Then, the edge of each pellet was selected using the “Wand” tool. A mask was then created to facilitate circularity analysis of the entire thin‐sliced pellet using the software function “Analyze Particle”.

## 3 Results and Discussion

The effects of the spore concentration in the inoculum (see Section 3.1) and the influences of added CaCl_2_ and Pluronic F68 (see Section 3.2) on the growth of *Penicillium* sp. (IBWF 040‐09) were investigated. Determination of biomass production (measured according to Section 2.3) and microscopic examination of thin‐sliced pellets (conducted according to Sections 2.7 and 2.8) were carried out. The level of protease‐inhibiting activity formed was determined by a protease inhibition test (see Section 2.5). The protease‐inhibiting activity of *Penicillium* sp. (IBWF 040‐09) has been studied since 2021 [21]. The chromatographic separation and purification of the corresponding substance are currently being optimized to enable a deeper investigation of its structure and inhibitory mechanism. In preliminary LC/MS studies, a protease inhibitor with a mass‐to‐charge ratio of 366 m/z was identified. The fully developed methods will be presented in an upcoming publication. Based on this knowledge, we use the term “protease inhibitor” in the following for the chromatographic fraction with protease‐inhibiting activity.

It is important to emphasize that both the quantity of substance produced and the reproducibility of the process must be taken into account when optimizing fungal cultivation. Furthermore, a toxicity test (see Section 2.6) was performed to evaluate the potential of the protease inhibitor as a drug candidate (see Section 3.3).

### 3.1 Spore Concentration in the Inoculum

**FIGURE 1:**
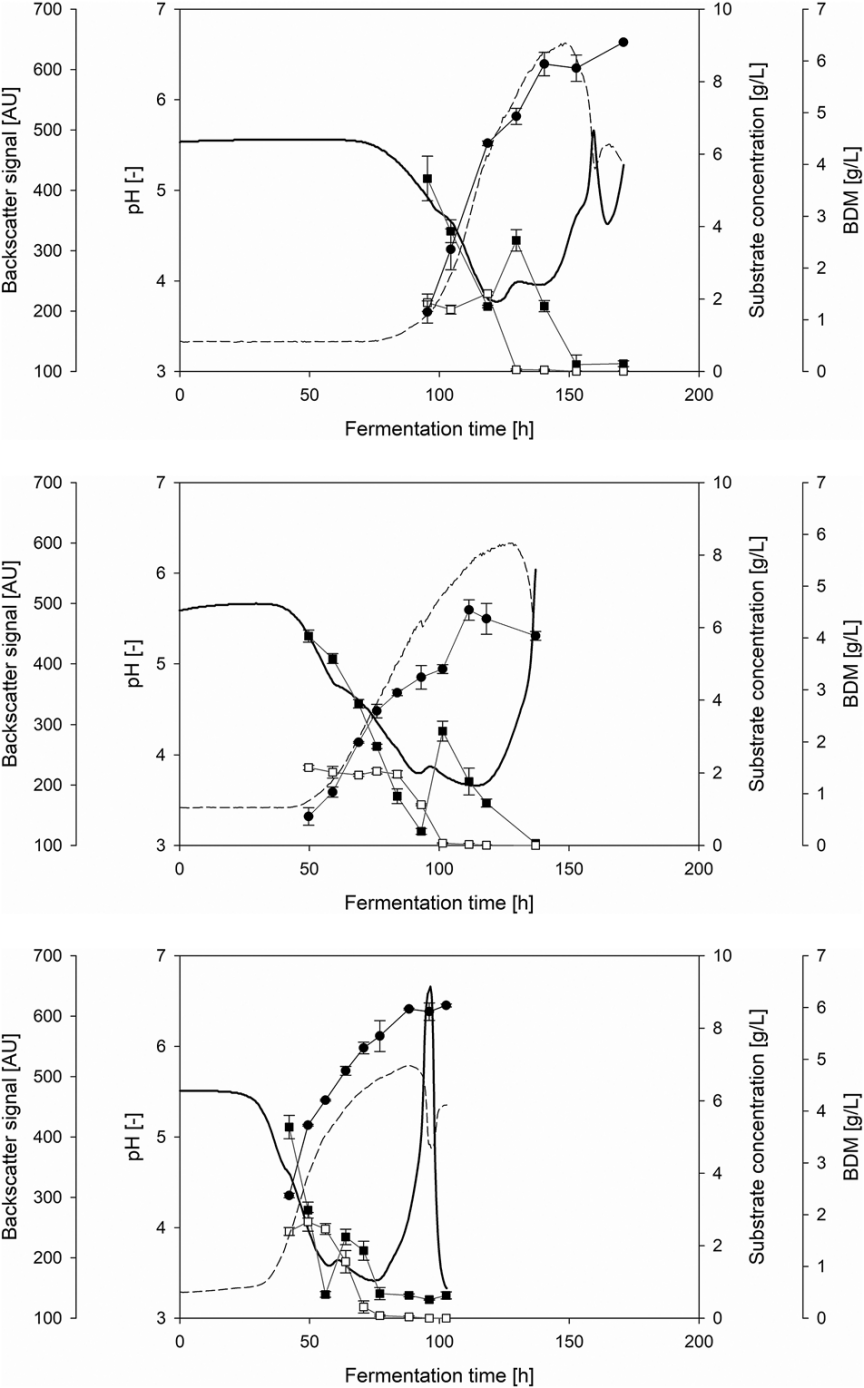
Cultivation courses of *Penicillium* sp. (IBWF 040‐09) in 2 L glass bioreactor (22°C, 350 rpm, initial aeration 1 L/min) with three different spore concentrations. Top: 4.41 · 10^3^ Sp/L; Middle: 4.41 · 10^5^ Sp/L; Bottom: 4.41 · 10^7^ Sp/L; 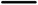: pH value; 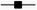: glucose conc.; 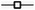: maltose conc.; 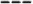: biomass conc. from backscatter sensor; 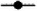: bio dry mass (BDM) conc.

Three different spore concentrations in the inoculum were investigated, and their influences on the cultivation process were observed. At least three individual cultivations were carried out for each spore concentration. The cultivation progress for each spore concentration is compared exemplarily in Figure 1. In general, the pH course of a fungal cultivation reflects the metabolic activity. In the present case with YMG medium, two decreases in the pH value were observed. The first decrease indicates the glucose consumption, whereas the second one reflects the maltose consumption [21, 25]. Similar fungal metabolic activity was demonstrated in the cultivations with different spore concentrations, as shown in Figure 1. However, the duration of the lag phase of each category differed. The longest lag phase of around 60 h was observed during the cultivation with the lowest spore concentration (i.e., 4.41 · 10^3^ Sp/L), whereas cultivation inoculated with the highest spore concentration at 4.41 · 10^7^ Sp/L led to a shorter lag phase of around 30 h.

The shorter lag phase at higher spore concentrations was most probably caused by the increased likelihood of spore–spore and spore–hyphae interactions during the aggregation process. This resulted in faster pellet formation. The same phenomenon was also acknowledged by Grimm et al. [13] during the spore aggregation of *A. niger*, classified as a coagulative type. The higher spore concentration led to faster second aggregation velocity; thus, the lag phase ended sooner. Since the pellets were formed earlier, the cultivation ended earlier as well. Accordingly, the cultivations with the highest spore concentration, 4.41 · 10^7^ Sp/L, were completed the earliest at 105 ± 16 h compared to 133 ± 5 h (+ 27 %) with 4.41 · 10^5^ Sp/L and 173 ± 7 h (+ 65 %) with 4.41 · 10^3^ Sp/L.

Regarding the biomass concentration (BDM), which was reached at the end of the cultivation, spore concentrations of 4.41 · 10^7^ Sp/L and 4.41 · 10^3^ Sp/L showed very similar average values at 5.53 ± 0.48 g/L and 5.24 ± 0.61 g/L, respectively, while 4.41 · 10^5^ Sp/L led to a slightly lower average BDM of 4.98 ± 0.64 g/L. This means that more or less the same biomass concentration (± 5 % for all cultivations) was achieved at the end of the cultivations with the three different spore concentrations. The small variation in biomass concentration observed in cultivations with different inoculum sizes contrasts with findings from several studies on filamentous microorganisms. For instance, Nielsen et al. [11] demonstrated a proportional correlation between spore concentration in the inoculum and the number of pellets formed by *P. chrysogenum*. Similarly, Abd‐Elsalam [26] reported increased biomass production in *A. ochraceus*, classified as non‐coagulative, with higher inoculum spore concentrations. This phenomenon appears to be independent of spore type, as *A. niger*, classified as a coagulative type, also exhibited the same proportional trend, as reported by Metz and Kossen [27].

The calculated growth rates also did not show any particular trend regarding the spore concentration: A spore concentration of 4.41 · 10^5^ Sp/L led to an average growth rate of 0.0684 ± 0.007 h^−1^, whereas spore concentrations of 4.41 · 10^3^ Sp/L and 4.41 · 10^7^ Sp/L exhibited similar average growth rates of 0.051 ± 0.004 h^−1^ and 0.0534 ± 0.020 h^−1^, respectively. These results indicate a minor effect of the spore concentration on growth rate during the exponential phase and biomass formation. In contrast, the spore concentration had a major influence on the duration of the lag phase and, thus, on the cultivation time. Assuming an equal ratio of product to biomass, this leads to correspondingly lower space‐time yields with longer cultivation times. From this point of view, a higher spore concentration, therefore, appears to be more suitable. However, it needs to be taken into consideration that the cultivations with a spore concentration of 4.41 · 10^7^ Sp/L showed poor reproducibility, with standard deviation of the average growth rate of 37 %, which is undesired.

In contrast to biomass concentration and growth rate, variations in spore concentration in the inoculum influenced pellet morphology in the submerged cultivation. The micromorphology of the pellets was examined on the basis of thin‐cuts produced using the paraffin‐embedding method (see Section 2.7). With an inoculum of 4.41 · 10^3^ Sp/L, the pellets were smooth on the outside with a thick edge, but the interiors were hollow, indicating pellet autolysis. Higher spore concentrations resulted in more “hairy” pellets with a porous inner structure and apparent hyphal distribution. Notably, more pellets from cultivations with an inoculum of 4.41 · 10^7^ Sp/L had intact cores. These results are in accordance with a study by Sharma and Padwal‐Desai [28] on *A. parasiticus* in shaking flasks. Here too, a smoother and more compact pellet morphology was observed in the cultivation with lower spore concentration at 10^2^ Sp/L, whereas 10^6^ Sp/L exhibited looser pellets.

**FIGURE 2:**
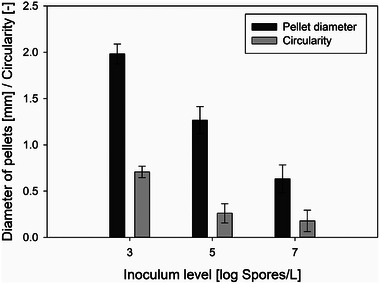
Morphology analysis of pellets from cultivations with different spore concentrations in the inoculum. All cultivations — at least three individual replicates of each category — were supplemented with 0.2 % (w/v) Pluronic F68 and 1.0 g/L CaCl_2_.

A notable correlation could be identified between spore concentration and pellet diameter, where lower spore concentration was associated with an increase in pellet size, as demonstrated in Figure 2. The largest pellet diameters of 1.98 ± 0.11 mm were observed in cultivations with an inoculum concentration of 4.41 · 10^3^ Sp/L. This was followed by pellets with diameters of 1.27 ± 0.15 mm, resulting from an inoculum concentration of 4.41 · 10^5^ Sp/L. The smallest pellet diameters of 0.63 ± 0.15 mm were produced in cultivations with the highest spore concentration of inoculum at 4.41 · 10^7^ Sp/L. A similar trend was observed by Sharma and Padwal‐Desai [28], where an increase in spore concentration from 10^2^ Sp/L to 10^6^ Sp/L resulted in a decrease in pellet size from 0.82 ± 0.13 mm to 0.33 ± 0.04 mm. Tucker and Thomas [29] and Papagianni and Mattey [10] reported a similar behavior of *P. chrysogenum* and *A. niger*, respectively. Contrary to the influence of the spore concentration on the outer pellet diameter, an increase in the spore concentration of the inoculum led to an increase in the pellet core diameter during the cultivation of *P. chrysogenum* in a study by Nielsen et al. [11].

The influence of spore concentration in the inoculum on pellet circularity was also investigated. As shown in Figure 2, pellet circularity decreased as the spore concentration increased. Pellets from cultivations with the lowest spore concentration exhibited the most circular shape, with circularity values of 0.707 ± 0.061, whereas the highest spore concentration led to the least circular pellets and more irregular shapes with values of 0.178 ± 0.115. Similarly, Tucker and Thomas [29] observed a more pronounced morphological shift, where an increase in spore concentration in the inoculum resulted in a transition from a pellet to a dispersed form.

**FIGURE 3:**
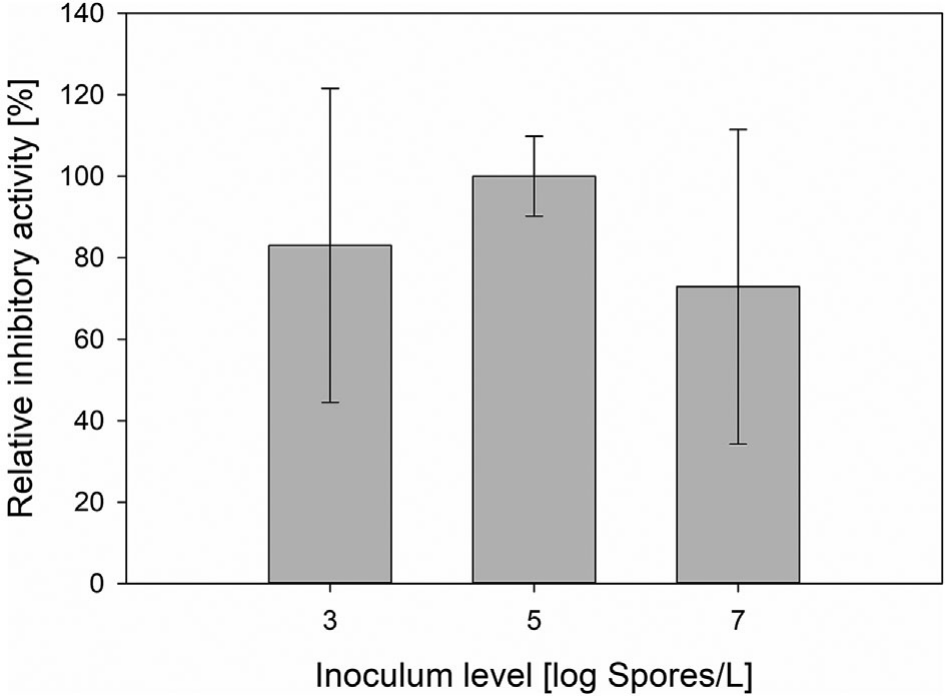
Relative inhibitory activities of B6 fractions from cultivations with three different spore concentrations in the inoculum. All cultivations — at least three individual replicates of each category — were supplemented with 0.2 % (w/v) Pluronic F68 and 1.0 g/L CaCl_2_.

Since the main objective of the cultivation of *Penicillium* sp. (IBWF 040‐09) is the production of the protease‐inhibiting substance, the influence of the spore concentration on the yield of the target substance also has to be investigated. A cultivation was considered complete when all provided substrates, glucose and maltose, were completely consumed (see Figure 1). Methanolic extract was prepared from fungal biomass and then fractionated into 92 fractions using HPLC, according to Section 2.4. The protease‐inhibiting activity of the B6 fraction contained 85 % purity of the target protease inhibitor based on preliminary studies (data not shown). The inhibitory activity of the B6 fraction, therefore, corresponded very well to the yield of the protease inhibitor. As a result, the inhibitory activity could be used to compare cultivations with different spore concentrations in the inoculum, as shown in Figure 3. Cultivations with a spore concentration of 4.41 · 10^5^ Sp/L resulted in the highest yield of protease inhibitor, with an average inhibitory activity of 92 ± 10 %, followed by 76 ± 39 % for the cultivations with 4.41 · 10^3^ Sp/L and 67 ± 39 % for 4.41 · 10^7^ Sp/L.

As a result, a spore concentration of 4.41 · 10^7^ Sp/L showed a 27 % shorter cultivation time, but, at the same time, it resulted in a 27 % lower yield of protease inhibitor combined with poor reproducibility compared to a spore concentration of 4.41 · 10^5^ Sp/L. The smaller diameters of hairy pellets produced in cultures with higher spore concentrations in the inoculum had probably reduced nutrient limitations within the pellets. As a consequence, there was a lower production of certain secondary metabolites, such as the specific protease inhibitor in the present case. A spore concentration of 4.41 · 10^3^ Sp/L was the least favorable in terms of both the yield of protease inhibitor and the cultivation time. With longer cultivation times, the shear forces caused by the stirrer had a correspondingly longer effect on the pellets formed. This resulted in increased shearing of hyphae from the pellets and increased growth in hyphae form. Although approximately the same mass of fungus was present at the end of the cultivation, the ratio of pellets to hyphae was reduced, and, therefore, less target substance was formed.

In the present case, the highest space‐time yield was achieved with a spore concentration of 4.41 · 10^5^ Sp/L. Therefore, this spore concentration is optimal for the production of the protease inhibitor by cultivating *Penicillium* sp. (IBWF 040‐09) in the bioreactor system used.

### 3.2 Addition of CaCl_2_ and Pluronic F68

**FIGURE 4:**
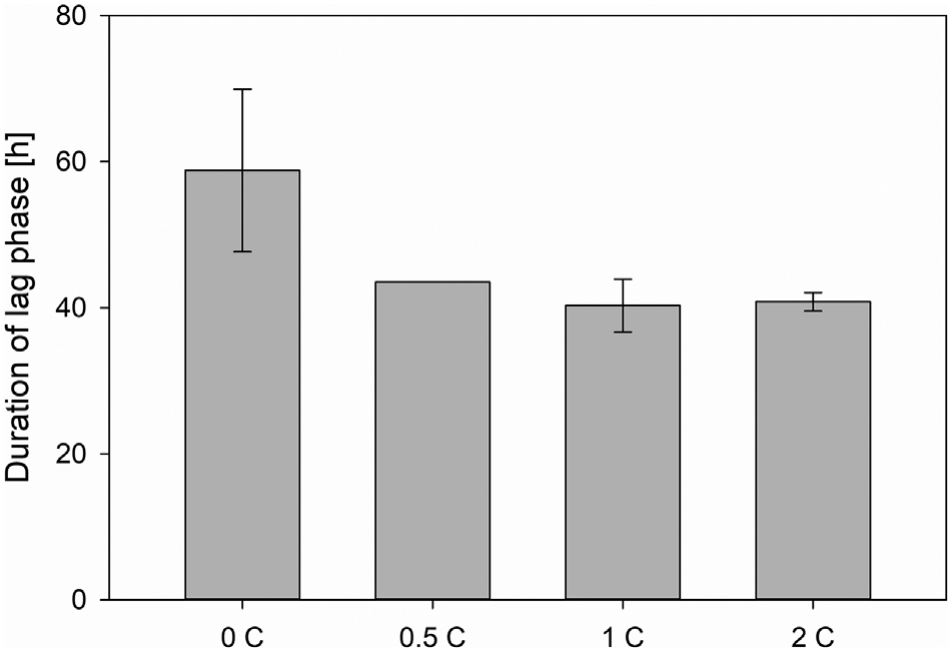
Comparison of durations of lag phases during cultivations, which were influenced by different concentrations of CaCl_2_ in medium (0C = 0 g/L, 0.5C = 0.5 g/L, 1C = 1.0 g/L, 2C = 2.0 g/L). All cultivations — at least three individual cultivations of each category — were started with 4.41 · 10^5^ Sp/L and 0.2 % (w/v) Pluronic F68.

In addition to spore concentration, medium composition also plays a key role in the cultivation processes of filamentous fungi, specifically in this study for the production of the protease inhibitor. The influence of both CaCl_2_ and Pluronic F68 in the cultivation medium on pellet morphology as well as on the formation of the protease inhibitor was investigated. For each variation, at least three individual cultivations were carried out. The effects of CaCl_2_ and Pluronic F68 on the fungal growth were investigated in two cultivation phases, namely, the lag phase and the exponential phase. The effect of different concentrations of CaCl_2_ in cultivation medium on the duration of the lag phase is shown in Figure 4.

The lag phase got shorter as the concentration of CaCl_2_ increased. Without CaCl_2_, the lag phase occurred for 58.8 ± 11.1 h. The addition of 0.5 g/L CaCl_2_ resulted in a shorter lag phase of 43.5 ± 0 h. This demonstrated the enhancement of spore aggregation processes, which led to faster pellet development. The result was in accordance with the theory about CaCl_2_ described by Braun and Vecht‐Liftshitz [16]. As a multivalent cation, Ca^2+^ supports the aggregation processes, as the cell walls of microorganisms are negatively charged at pH values ≥ 5.5. However, increasing the CaCl_2_ concentration from 0.5 g/L to 2.0 g/L did not lead to a further significant shortening of the lag phase.

**FIGURE 5:**
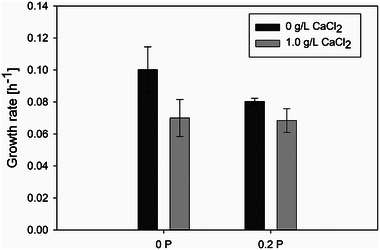
Comparison of fungal growth rates of cultivations with different additions of Pluronic F68 (0P = 0 % (w/v), 0.2P = 0.2 % (w/v)) and CaCl_2_. All cultivations — at least three individual cultivations of each category — were started with a spore concentration of 4.41 · 10^5^ Sp/L.

While CaCl_2_ was observed to influence the lag phase, the addition of Pluronic F68 into the cultivation medium exhibited effects on fungal growth during the exponential phase (see Figure 5). These results indicated that the addition of Pluronic F68 reduced the growth rate. Pluronic F68 is known to reduce the shear stress caused by the bursting of air bubbles by stabilizing them [20]. The lower shear stress allows the growing pellets to retain their pellet shape better. As a result, fewer hyphae are shed from the pellets, and fewer clumps that grow in the form of hyphae are formed in the cultivation medium. On the other hand, without Pluronic F68, the fungal pellets were exposed to higher shear stress, which tend to break the pellet form. The hyphae released from the broken pellets grow into new clumps. The slower growth rate of fungi in the pellet form in comparison to the dispersed form was also reported by Pera and Callieri [17] and Vecht‐Lifshitz et al. [18]. The effect of Pluronic F68 on the growth rate was, however, significantly more pronounced in the absence of CaCl_2_ in the cultivation medium (see Figure 5). In the presence of CaCl_2_, the positive effect of Pluronic F68 in the exponential phase was hardly significant. This again showed the positive influence of CaCl_2_ on the aggregation processes in the run‐up to pellet growth.

**FIGURE 6:**
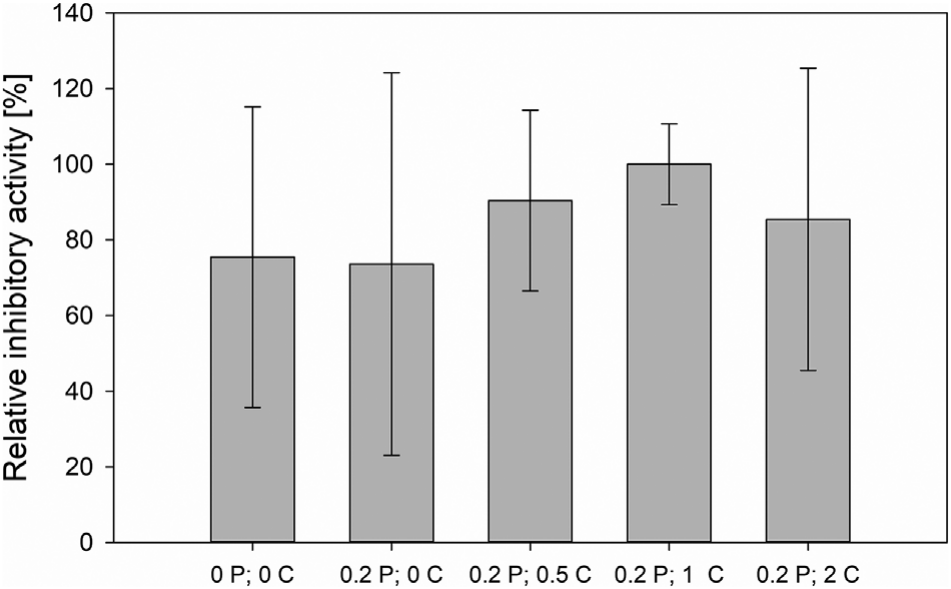
Comparison of the relative protease‐inhibiting activities in the B6 fractions of cultivations — at least three individual cultivations of each category — with Pluronic F68 (0.2P = 0.2 % (w/v)) and various concentrations of CaCl_2_ (0C = 0 g/L, 0.5C = 0.5 g/L, 1C = 1.0 g/L, 2C = 2.0 g/L). Cultivations without Pluronic F68 (0P = 0 % (w/v)) and CaCl_2_ were used as the reference.

The addition of CaCl_2_ and Pluronic F68 was also investigated with regard to the yield of the protease inhibitor, which is represented by the inhibitory activity of the B6 fraction. Figure 6 shows the relative protease‐inhibiting activity of the B6 fractions of fungal extracts from cultivations with various medium compositions. As a control, cultivations were also carried out without the addition of Pluronic F68 and CaCl_2_. These resulted in a relative inhibition activity of 75 %, with a high standard deviation of around 40 %, which indicates poor reproducibility. This means that there is a high probability that cultivations without both substances will result in undesired low production of the protease inhibitor. It is noteworthy that the lowest standard deviation of only 10 % in the tests performed was associated with the highest inhibition activity. This is the case when 0.2 % (w/v) Pluronic F68 and 1.0 g/L CaCl_2_ were added to the cultivation medium (see Figure 6).

**FIGURE 7:**
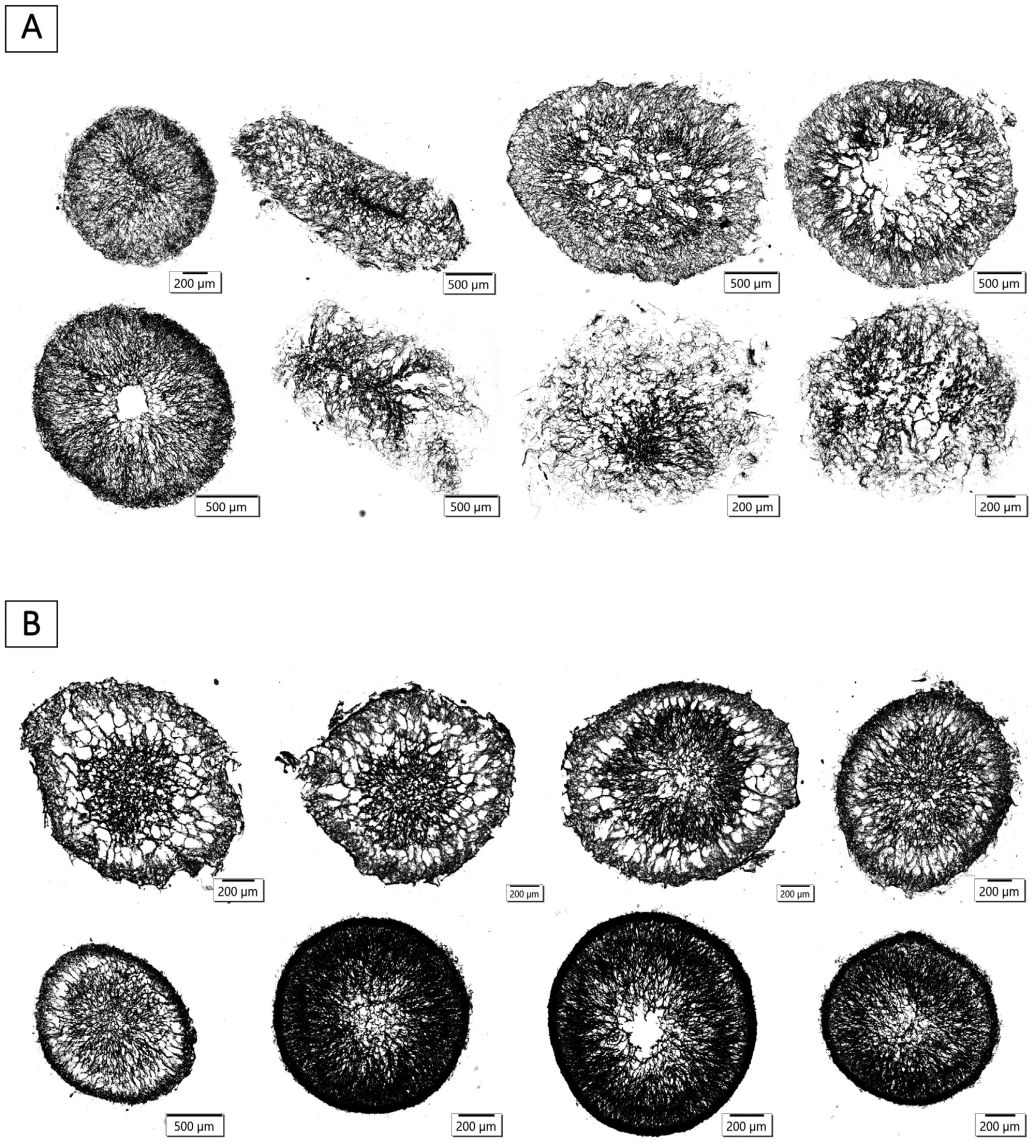
Various images showing the micromorphology of pellets from cultivations without CaCl_2_ and Pluronic F68 (A) and cultivations with 1.0 g/L CaCl_2_ and 0.2 % (w/v) Pluronic F68 (B).

Since CaCl_2_ and Pluronic F68 play important roles in the aggregation processes (lag phase) and the preservation of the pellet shape (exponential phase), they should also have an influence on the pellet structure. The micromorphology of the pellets was, therefore, also investigated in this study. A comparison of the morphology was made between cultivations with and without both substances (see Figure 7).

Without the addition of CaCl_2_ and Pluronic F68, the pellets tended to be fluffy, and the hyphae appeared to be more evenly distributed over the pellets (see Figure 7A). On the contrary, the morphology of the pellets from the cultivations with CaCl_2_ and Pluronic F68 showed an overall denser structure. There were clearly different layers with more or less densely distributed hyphae. In some cases, lysis of the pellet core also occurred (see Figure 7). Based on the results of the inhibitory activity tests, the denser pellets led to a higher yield of the target substance. Overall, the medium composition with 1.0 g/L CaCl_2_ and 0.2 % (w/v) Pluronic F68 seemed to be optimal for the production of protease inhibitor with *Penicillium* sp. (IBWF 040‐09). However, it should be noted that the reproducibility of the fungal pellet morphology was generally low. This applied even under exactly the same cultivation conditions and is due to different spore batches. Hille et al. [30] demonstrated that cultivations resulted in two completely different pellet shapes with different hyphae distribution and oxygen profiles when working with different spore batches under otherwise identical conditions. Based on this fact, adjusting the composition of the medium can only increase the probability of achieving the desired pellet morphology and yield of the target substance.

### 3.3 Cytotoxicity of the Protease Inhibitor

The development of new drugs only makes sense if the discovered compound is not toxic to human cells. Early cytotoxicity tests are, therefore, essential before starting cellular assays and in vivo studies with the drug candidate. This also applies to the protease inhibitor produced by *Penicillium* sp. (IBWF 040‐09) as a potential future drug against HAT. To carry out cytotoxicity tests with PBMCs, the corresponding fraction from the preparative HPLC (see Section 2.4) was used. The preparative fraction also contained other substances that could also be cytotoxic because the separation of the target substance with preparative HPLC was not as optimal as the separation with analytical HPLC.

**FIGURE 8:**
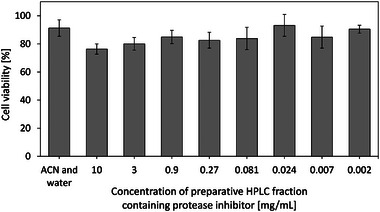
Resazurin assays were performed on peripheral blood mononuclear cells treated with different concentrations of the fraction of preparative HPLC containing protease inhibitor. Data are expressed as mean values ± standard deviations from three experiments. The mixture of acetonitrile (ACN) and water was used as the negative control of the assay.

The cell viability of PBMCs exposed to different concentrations of the corresponding fraction was compared with the cell viability of PBMCs exposed to an ACN–water mixture as a negative control. Based on the results of the resazurin assays shown in Figure 8, the cell viability of PBMCs was higher than 76 % in all concentrations. The comparison with the reference with approximately 90 % cell viability shows that the examined fraction did not appear to have a cytotoxic effect on PBMCs. Because this whole fraction was found not to be cytotoxic, it can be concluded that the pure target substance, which is the major component of the fraction, is also non‐toxic.

## 4 Concluding Remarks

The bioreactor cultivation of *Penicillium* sp. (IBWF 040‐09), which produces a protease‐inhibiting substance, was optimized to achieve a high yield of the target substance in combination with good reproducibility. Therefore, the medium composition, in this case, the addition of both Pluronic F68 and CaCl_2_, as well as the spore concentration, was varied. The cultivations with higher spore concentrations ended earlier than those with lower spore concentrations due to a shorter lag phase. Moreover, higher spore concentration in the inoculum led to smaller pellets with a more irregular internal structure. The addition of CaCl_2_ supported the pellet formation during the lag phase, whereas Pluronic F68 helped maintain the pellet shape during the exponential phase. A spore concentration of 4.41 · 10^5^ Sp/L and the addition of 0.2 % Pluronic F68 and 1.0 g/L CaCl_2_ were determined to be the optimal conditions for the cultivation of *Penicillium* sp. (IBWF 040‐09) in the bioreactor setup used. With these parameters, cultivations with good reproducible formation of the protease inhibitor are possible, which can be deduced from the achieved inhibitory activity (with a standard deviation < 10 %).

As the protease‐inhibiting substance did not show noticeable cytotoxicity toward human blood cells, there is a chance that a new drug can be developed. The structural elucidation of the substance is, therefore, currently being driven forward. The inhibition mechanism also needs to be investigated to better understand this protease inhibitor and its potential as a drug against HAT. The corresponding results will be presented in an upcoming publication.

## Conflicts of Interest

The authors declare no conflicts of interest.

## Data Availability

The data that support the findings of this study are available from the corresponding author upon reasonable request.
